# The Mediating Role of Psychological Capital on the Association between Occupational Stress and Job Burnout among Bank Employees in China

**DOI:** 10.3390/ijerph120302984

**Published:** 2015-03-10

**Authors:** Xirui Li, Dan Kan, Li Liu, Meng Shi, Yang Wang, Xiaoshi Yang, Jiana Wang, Lie Wang, Hui Wu

**Affiliations:** 1 Department of Social Medicine, School of Public Health, China Medical University, No.77 Puhe Road, Shenyang North New Area, Shenyang 110013, China; E-Mails: lixirui1004@sina.cn (X.L.); liul@mail.cmu.edu.cn (L.L.); wangyang201533@163.com (Y.W.); yangxiaoshi@mail.cmu.edu.cn (X.Y.); jiana0818@163.com (J.W.); liewang@mail.cmu.edu.cn (L.W.); 2 The First Hospital of China Medical University, No. 155 Nanjingbei Road, Heping District, Shenyang 110001, China; E-Mail: kandan30@163.com; 3 Department of English, China Medical University, No.77 Puhe Road, Shenyang North New Area, Shenyang, China; E-Mail: 18900910948@163.com

**Keywords:** job burnout, occupational stress, psychological capital, bank employees

## Abstract

Although job burnout is common among bank employees, few studies have explored positive resources for combating burnout in this population. This study aims to explore the relationship between occupational stress and job burnout among Chinese bank employees, and particularly the mediating role of psychological capital. A cross-sectional study was conducted in Liaoning, China, during June to August of 2013. A questionnaire that included the effort-reward imbalance scale, the Psychological Capital Questionnaire and the Maslach Burnout Inventory-General Survey, as well as demographic and working factors, was distributed to 1739 employees of state-owned banks. This yielded 1239 effective respondents (467 men, 772 women). Asymptotic and resampling strategies explored the mediating role of psychological capital in the relationship between occupational stress and job burnout. Both extrinsic effort and overcommitment were positively associated with emotional exhaustion and depersonalisation. Meanwhile, reward was negatively associated with emotional exhaustion and depersonalisation, but positively associated with personal accomplishment. There was a gender difference in the mediating role of Psychological capital on the occupational stress-job burnout. In male bank employees, Psychological capital mediated the relationships of extrinsic effort and reward with emotional exhaustion and depersonalization; in female bank employees, it partially mediated the relationships of extrinsic effort, reward and overcommitment with emotional exhaustion and depersonalisation, as well as the relationship between reward and personal accomplishment. Psychological capital was generally a mediator between occupational stress and job burnout among Chinese bank employees. Psychological capital may be a potential positive resource in reducing the negative effects of occupational stress on job burnout and relieving job burnout among bank employees, especially female bank employees.

## 1. Introduction

Job burnout (burnout) is an individual reaction to emotional and interpersonal stress and is related to work pressure and occupational stress [1]. Burnout comprises the three dimensions of emotional exhaustion, depersonalisation and personal accomplishment [2]. Emotional exhaustion is a negative state of excessive emotional consumption and lack of energy. Depersonalisation is a negative, indifferent and excessively distant attitude to service objects. Personal accomplishment is a feeling of competence and successful achievement in one’s work [3].

Originating in America in the 1970s, job burnout studies have nearly 40 years of history [3]. As social reforms have progressed and competitive pressures have increased, the highly specific occupational hazard of job burnout has attracted growing attention [4,5,6]. Burnout affects both physical and mental health, and symptoms can include chronic fatigue, headaches, depression and anxiety [7,8,9,10]. Burnout can also impact employees’ job performance and involvement [7,11]. Previous studies often discussed burnout separately from its three dimensions [10,12,13], and found different levels of burnout not only in teachers, policemen, physicians and nurses, but also in bank employees [7,14,15]. State-owned banks have recently experienced shareholding and system reform, increasing the work pressure faced by bank employees. Because banks are service businesses, bank jobs can involve significant interpersonal pressure, which may lead to long-term energy consumption among bank employees. Failure to effectively control this pressure will eventually produce job burnout. Thus, it is important to study the status and causes of job burnout, both to reduce the phenomenon and to prevent it affecting bank employees’ work.

Occupational stress was identified as a risk factor of job burnout in previous studies, and was measured using the effort–reward imbalance (ERI) model [16,17,18]. With its focus on individual vital interests at work, the ERI model may be particularly suitable for studying the adverse health effects of occupational stress as currently witnessed in China. According to research on stress, the risk of burnout caused by occupational stress was seven times higher under high occupational stress than low occupational stress [19,20]. High extrinsic effort and overcommitment predicted the increase of emotional exhaustion and depersonalisation, and the decline of personal accomplishment, both of which contradicted the role of reward [4,21]. As their job content is connected to finance, bank employees must maintain high concentration. Bank employees need pay particular attention to their emotional expression when they communicating directly with customers [22,23]. Bank employees thus become a susceptible and high-risk population of occupational stress. According to previous studies, occupational stress both directly and indirectly affects all the dimensions of job burnout [18,24,25]. However, we are aware of few studies that have examined both effects of occupational stress on job burnout among bank staff.

During recent years, scholars have begun to explain the mechanism of job burnout from the resource perspective. Psychological capital (PsyCap) is an important personal resource, defined by Luthans *et al.* as “a positive psychological state that an individual performs in the process of growth and development” [26]. It is composed of four state-like psychological resource capacities, namely self-efficacy, hope, optimism and resilience, all of which can be measured, developed, and effectively managed with various desirable results [27]. Specifically, employees with high PsyCap, possess extra resources to handle their work tasks, expect good things to happen, quickly “bounce back” from setbacks, and are more optimistic about negative situations.

According to previous reports, PsyCap significantly and positively affected employees’ job satisfaction, job performance, well-being, organizational commitment and quality of life [28,29,30,31,32,33]. PsyCap is also a positive resource able to prevent stress, turnover, anxiety and depression [34,35,36]. Additionally, PsyCap has been identified as a mediator in the relationship between supportive organizational climate and employee performance [37]. Wang *et al.* reported that PsyCap mediated the relationship between work-family conflict and the three dimensions of burnout among Chinese doctors and nurses [14,38]. Although the association between occupational stress and job burnout or between PsyCap and job burnout has been confirmed in various professions [14,16,38], the mediating role of PsyCap between occupational stress and job burnout has not been examined among bank employees to our knowledge. To fill this gap, this study examines the potential mediating effect of PsyCap on the posited association between occupational stress and job burnout, focusing on a Chinese population.

Furthermore, gender differences have been observed in the correlations between occupational stress and related outcomes [39,40]. For instance, Song *et al.* found male individuals to have better mental health than female individuals [39]. The Chinese burnout index survey also showed higher job burnout in women than men. Traditional Chinese culture associates different social expectations and household responsibilities with different gender roles. Therefore, we examined the association between occupational stress and job burnout separately in male and female bank employees to investigate the role of gender as a moderator in the posited relationships.

Based on the above analysis, the purposes of this study are, separately for both male and female bank employees: (1) to examine the association of occupational stress and job burnout, (2) to determine the association of PsyCap and job burnout, and (3) to explore whether PsyCap mediates the association between occupational stress and job burnout among Chinese bank employees.

## 2. Methods

### 2.1. Study Design and Sample

A cross-sectional survey was conducted in Liaoning province (population: 43 million), northeast China, from June to August 2013. According to the geographic distribution of Liaoning province, one city was randomly selected from each region (eastern, western, southern, northern and central). Two large state-owned banks were further randomly selected from each city. A total of 10 state-owned banks were thus selected from five cities. We randomly sampled 50% of the employees from each bank. Self-administered questionnaires were directly distributed to 1739 employees after obtaining their written informed consent. Complete responses were obtained from 1239 individuals, including 467 (37.7%) male individuals and 772 (62.3%) female individuals. The study protocol was approved by the ethical standards of the Committee on Human Experimentation of China Medical University.

### 2.2. Demographic and Working Characteristics

Demographic and working factors included age, marital status, education, monthly income, job seniority and weekly hours worked. Marital status was categorized as ‘single/widowed/divorced/separated’ and ‘married/cohabiting’. Education was categorized as ‘junior college or lower’, ‘college’, and ‘graduate or higher’. Monthly income was categorized as ‘≤3000 RMB’ or ‘>3000 RMB’. Job seniority was categorized as ‘head bank employee’ or ‘ordinary bank employee’ based on responses to the question ‘Are you a head bank employee or an ordinary bank employee?’ Weekly hours worked was categorized as ‘≤40 h’ and ‘>40 h’, a categorization derived from the current standard work system of 8 h per day.

### 2.3. Measurement of Occupational Stress

Occupational stress was assessed using the Chinese version of the ERI questionnaire for claiming failed reciprocity in terms of high efforts and low rewards [41]. The questionnaire comprises three subscales: extrinsic effort (six items), reward (eleven items) and overcommitment (six items). Each response for extrinsic effort and reward is scored from 1 to 5, and higher total scores indicate higher demands in terms of efforts and higher associated rewards. Responses for overcommitment are scored on a scale that ranges from 1, representing complete disagreement, to 4, representing complete agreement. A higher score suggests higher demands characterized by excessive work-related commitment. The Chinese version of the ERI scale has been widely applied among Chinese occupational groups and found to have good reliability and validity [42,43]. In this study, the Cronbach’s alphas for the extrinsic effort, reward and overcommitment subscales were 0.894, 0.864, and 0.729 for male bank employees and 0.886, 0.893, and 0.796 for female bank employees, respectively.

### 2.4. Measurement of Psychological Capital

The Chinese version of the 24-item Psychological Capital Questionnaire (PCQ) was used to measure PsyCap [44]. The PCQ consists of four subscales: self-efficacy (six items), hope (six items), resilience (six items) and optimism (six items). Each item is scored on a Likert scale where 1 indicates strong disagreement and 6 indicates strong agreement. All questions ask the participants how they feel “right now.” Because PsyCap is a higher-order core construct, the four key psychological resource capacities have a synergistic effect [37]. In this study, the average score for the total scale was calculated to obtain a composite PsyCap value, with higher scores indicating more PsyCap.

The Chinese version of the PCQ-24 was initially applied to a Chinese population, including company employees, college students and nurses [14,30,38,45]. The reliability and validity of the PCQ-24 questionnaire have been demonstrated in previous research [33,41,42]. For the total scale, the Cronbach’s alpha was 0.935 for male bank employees and 0.917 for female bank employees.

### 2.5. Measurement of Job Burnout

Job burnout was measured using the Chinese version of the Maslach Burnout Inventory-General Survey (MBI-GS), which has been widely applied across different occupational groups [46,47,48]. The 16-item MBI-GS questionnaire includes three dimensions: emotional exhaustion (EE, five items), depersonalisation (DE, five items) and personal accomplishment (PA, six items). For each item, there are seven possible answers: never, rarely (several times per year), sometimes (once a month), often (several times per month), frequently (once a week), always (several times per week) and every day. These responses are scored according to how often the statement is experienced, from 0 to 6. High scores on the emotional exhaustion or depersonalisation dimensions indicate burnout, as do low scores on the reduced personal accomplishment dimension. Wu *et al.* demonstrated good reliability and validity for the Chinese version of MBI when applied to a Chinese sample population [4]. In our study, the Cronbach’s alpha of the MBI-GS was 0.910. Meanwhile, the Cronbach’s alphas for the subscales of “emotional exhaustion”, “depersonalisation” and “personal accomplishment” were 0.960, 0.833 and 0.943 for male bank employees and 0.947, 0.787 and 0.933 for female bank employees.

### 2.6. Statistical Analysis

Pearson’s Chi-square (χ^2^) tests were used to compare differences in demographic characteristics and work conditions between male and female employees. Study variables were compared among age and education groups by one-way ANOVA analyses. T-tests were performed to examine the differences in continuous variables between male and female individuals.

**Figure 1:**
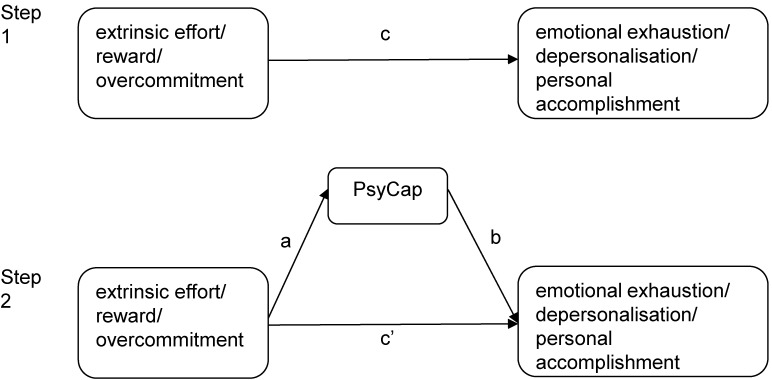
Theoretical model of the mediating role of psychological capital on the association between occupational stress and depressive symptoms. c: associations of extrinsic effort, reward or overcommitment with emotional exhaustion, depersonalisation or personal accomplishment; a: associations of extrinsic effort, reward or overcommitment with PsyCap; b: association between PsyCap and emotional exhaustion, depersonalisation or personal accomplishment after controlling for the predictor variables; c’: associations of extrinsic effort, reward or overcommitment with emotional exhaustion, depersonalisation or personal accomplishment after adding PsyCap as a mediator.

All the continuous variables were standardized to avoid multicollinearity. Additionally, two measures, tolerance and variance inflation factor, were used to check for multicollinearity. Pearson’s correlation coefficients were used to examine correlations among continuous variables. We used the asymptotic and resampling strategies developed by Preacher and Hayes to examine PsyCap as a potential mediator of the association between occupational stress evaluated by the ERI scale and job burnout [49]. In the regression equation, extrinsic effort, reward or overcommitment were modeled as predictors, with emotional exhaustion, depersonalisation or personal accomplishment as the outcomes, PsyCap as the mediator (as shown in Figure 1), and age, marital status, education and weekly hours worked as covariates. The first step in the analysis was to determine the association between each dimension of occupational stress and each dimension of job burnout (the c path) and the second was to estimate the mediating role (the a × b products) of PsyCap. When the c’ path coefficient in the second step was smaller than the c path coefficient in the first step, or was insignificant, it was speculated that mediation may exist. The bootstrap estimate presented in our study was based on 5000 bootstrap samples. A bias-corrected and accelerated 95% confidence interval (BCa 95% CI) was determined for each a × b product, and a BCa 95% CI other than 0 indicated significant mediation.

In all analyses, male and female employees were analyzed separately because of possible gender differences. All of the above analyses were conducted using SPSS 13.0 for Windows. Statistical significance was set at *p* < 0.05.

## 3. Results

### 3.1. Participant Characteristics

Demographic and working characteristics of the study subjects were shown separately for male and female bank employees in Table 1. The means and standard deviations of PA were 18.06 ± 11.09 and 20.88 ± 10.71 respectively for male and female bank employees, demonstrating significant difference. Male individuals obtained higher PA scores than female individuals owing to their having more opportunities to express their self-ability. Mean EE differed significantly between groups with different weekly hours worked for both male and female bank employees. Both male and female individuals working more than 40 h per week show higher EE, because they spend too long working and hence are more likely to feel bored and fatigued. Mean DE differed across marital status groups and weekly working hour groups only for female bank employees. To female bank employees, married or cohabiting statuses increased their obligations to care for either older relatives or their children but longer working time made it difficult to meet these obligations, potentially leading to poor service. Mean PA significantly differed across age and education groups for both male and female bank employees. Male bank employees who are aged 30–40 years or have higher educational level (graduate or above), could derive benefits based on their higher educational level and more enterprising nature. Meanwhile, female bank employees, who are aged under 30 years or have higher education level (graduate or above), often have high career expectations and confront working problems positively. Both groups thus have more opportunities for personal accomplishment.

### 3.2. Correlations between Study Variables

Correlations between the study variables are presented in Table 2. Age was negatively correlated with reward and PA for both males and female individuals, and was negatively correlated with PsyCap for male individuals. Age was also positively correlated with extrinsic effort for female individuals.

Two dimensions of job burnout (EE and DE) were positively correlated with extrinsic effort and overcommitment, and negatively correlated with reward and PsyCap. PA was positively correlated with reward and PsyCap. However, no significant correlations existed between either extrinsic effort or overcommitment and PA.

### 3.3. Regression Analysis with Emotional Exhaustion as the Criterion Variable

Path coefficients, a × b products and BCa 95% CI for these products were presented in Table 3. First, the associations between occupational stress evaluated by the ERI scale and emotional exhaustion (the c path) were determined. Positive associations of extrinsic effort and overcommitment with emotional exhaustion and a negative association of reward with emotional exhaustion were observed among both male and female bank employees. The mediating role of PsyCap in the association between occupational stress and emotional exhaustion was then estimated.

For male bank employees, extrinsic effort was negatively and reward was positively significantly associated with PsyCap, but overcommitment was not significantly associated with it (the a path). After controlling for the predictor variables, PsyCap was significantly and negatively associated with emotional exhaustion (the b path). Thus, PsyCap significantly mediated the associations of extrinsic effort and reward with emotional exhaustion. When PsyCap was included in the model as a mediator, the direct pathway between extrinsic effort and emotional exhaustion remained significant while that between reward and emotional exhaustion did not (the c’ path).

**Table 1:** Participant characteristics, means and standard deviations (SDs) of variables.

Variable	Males				Females			
	N (%)	EE	DE	PA	N (%)	EE	DE	PA
		Mean(SD)	Mean(SD)	Mean(SD)		Mean(SD)	Mean(SD)	Mean(SD)
Total	467 (37.7%)	8.29 (6.56)	8.75 (6.51)	18.06 (11.09)	772 (62.3%)	8.80 (6.47)	9.31 (6.19)	20.88 (10.71) **
Age (years)								
≤30	177 (37.9%)	8.12 (6.68)	8.64 (6.42)	18.86 (10.98)	383 (49.6%)	8.91 (6.72)	9.18 (6.19)	21.39 (10.39) *
30–40	137 (29.3%)	9.23 (6.06)	8.88 (6.06)	19.13 (10.54) *	237 (30.7%)	9.05 (6.30)	9.19 (5.92)	21.33 (10.64)
≥40	153 (32.8%)	7.64 (6.81)	8.77 (7.04)	16.18 (11.53)	152 (19.7%)	8.16 (6.09)	9.82 (6.62)	18.88 (11.41)
Marital status								
Single/widowed/divorced	160 (34.3%)	8.36 (6.90)	8.78 (6.56)	18.43 (10.99)	276 (35.8%)	8.22 (6.06)	8.62 (5.74)	21.35 (10.17)
Married/cohabiting	307 (55.7%)	8.25 (6.39)	8.74 (6.50)	17.87 (11.16)	496 (64.2%)	9.13 (6.67)	9.69 (6.40) *	20.61 (11.00)
Education								
Junior college or lower	54 (11.6%)	7.44 (6.79)	7.78 (6.40)	12.61 (9.76)	72 (9.3%)	7.92 (6.05)	8.74 (6.46)	14.72 (10.16)
College	338 (72.4%)	8.45 (6.69)	8.89 (6.55)	18.04 (10.89)	550 (71.2%)	9.01 (6.52)	9.49 (6.15)	21.35 (10.74)
Graduate or higher	75 (16.1%)	8.17 (5.81)	8.83 (6.47)	22.08 (11.32) **	150 (19.4%)	8.48 (6.48)	8.91 (6.22)	22.09 (9.93) **
Monthly income (RMB)								
≤3000	178 (38.1%)	8.60 (6.75)	9.13 (6.79)	17.70 (10.79)	327 (42.4%)	9.03 (6.65)	9.78 (6.09)	20.96 (10.63)
>3000	259 (61.9%)	8.09 (6.45)	8.52 (6.33)	18.29 (11.29)	445 (57.6%)	8.64 (6.34)	8.96 (6.25)	20.82 (10.77)
Job seniority								
Head bank employee	99 (21.2%)	8.37 (5.80)	7.99 (5.65)	19.17 (11.54)	126 (16.3%)	9.68 (6.06)	9.72 (6.20)	22.14 (10.27)
Common bank employee	368 (78.8%)	8.26 (6.76)	8.96 (6.72)	17.76 (10.96)	646 (83.7%)	8.63 (6.54)	9.23 (6.19)	20.63 (10.78)
Weekly hours worked								
≤40 h	165 (35.3%)	6.96 (6.36)	8.24 (5.69)	17.69 (11.63)	242 (31.3%)	7.32 (5.49)	8.63 (5.60)	21.03 (10.86)
>40 h	302 (64.7%)	9.01 (6.57) **	9.03 (6.42)	18.26 (10.80)	530 (68.7%)	9.48 (6.77) **	9.62 (6.43) *	20.81 (10.65)

Notes: EE: Emotional exhaustion; DE: Depersonalisation; PA: Personal accomplishment. * significant at the 0.05 level (2-tailed); ** significant at the 0.01 level (2-tailed).

**Table 2:** Means, standard deviations (SD) and correlations of all variables.

Variable	Males				Females			
	1	2	3	4	5	6	7	8
1. Age(yr)	-	0.085 *	−0.237 **	0.026	−0.067	−0.002	0.057	−0.106 **
2. Extrinsic effort	−0.035	-	−0.229 **	0.361 **	−0.297 **	0.494 **	0.318 **	−0.015
3. Reward	−0.216 **	−0.229 **	-	−0.161 **	0.256 **	−0.181 **	−0.174 **	0.220 **
4.Overcommitment	0.018	0.337 **	−0.126 **	-	−0.178 **	0.282 **	0.139 **	−0.031
5. PsyCap	−0.094 *	−0.192 **	0.219 **	−0.038	-	−0.363 **	−0.270 **	0.187 **
6. EE	−0.024	0.458 **	−0.127 **	0.250 **	−0.300 **	-	0.649 **	0.147 **
7. DE	0.025	0.326 **	−0.135 **	0.145 **	−0.263 **	0.740 **	-	0.354 **
8. PA	−0.116 *	0.063	0.175 **	0.022	0.112 *	0.318 **	0.464 **	-

Notes: PsyCap: Psychological capital; EE: Emotional exhaustion; DE: Depersonalisation; PA: Personal accomplishment. * significant at the 0.05 level (2-tailed); ** significant at the 0.01 level (2-tailed).

**Table 3:** Regression analysis results, with emotional exhaustion as outcome and psychological capital as mediator.

Predictors	Path Coefficients				a × b (BCa 95% CI)	R^2^
	c	a	b	c’		
Males						
Extrinsic effort	0.456 **	‒0.228 **	‒0.213 **	0.408 **	0.048 (0.025, 0.082)	0.246
Reward	‒0.142 **	0.223 **	‒0.282 **	‒0.079	‒0.065 (‒0.100, ‒0.033)	0.093
Overcommitment	0.266 **	‒0.056	‒0.286 **	0.250 **	0.016 (‒0.013, 0.048)	0.143
Females						
Extrinsic effort	0.496 **	−0.286 **	‒0.251 **	0.425 **	0.073 (0.046, 0.098)	0.295
Reward	‒0.191 **	0.237 **	‒0.351 **	‒0.108 **	‒0.083 (‒0.119, ‒0.057)	0.147
Overcommitment	0.265 **	‒0.162 **	‒0.338 **	0.210 **	0.056 (0.028, 0.083)	0.183

Notes: c: associations of extrinsic effort, reward and overcommitment with emotional exhaustion; a: associations of extrinsic effort, reward and overcommitment with PsyCap; b: association between PsyCap and emotional exhaustion after controlling for the predictor variables; c’: associations of extrinsic effort, reward and overcommitment with emotional exhaustion after adding PsyCap as mediator; a × b: the product of a and b; BCa 95% CI: the bias-corrected and accelerated 95% confidence interval age, marital status, education and weekly work time are covariates; * significant at the 0.05 level (2-tailed); ** significant at the 0.01 level (2-tailed).

For female bank employees, extrinsic effort and overcommitment were negatively and reward was positively associated with PsyCap (the a path). As for the results of male bank employees, PsyCap was significantly and negatively associated with emotional exhaustion after controlling for extrinsic effort, reward and overcommitment. Thus, PsyCap significantly mediated the associations of extrinsic effort, reward and overcommitment with emotional exhaustion. The direct pathways between extrinsic effort, reward, overcommitment and emotional exhaustion (the c’ path) remained significant when PsyCap was included in the model as a mediator.

To estimate the effect size of the mediating pathway, we calculated the proportion of the total effect of the independent variable on the dependent variable (c) that was mediated by PsyCap using the formula (a × b)/c. For male bank employees, the proportion of PsyCap mediation was 10.65% for extrinsic effort, and 44.29% for reward. For female bank employees, the proportions of PsyCap mediation were 14.47% for extrinsic effort, 43.55% for reward and 20.66% for overcommitment.

### 3.4. Regression Analysis with Depersonalisation as the Criterion Variable

Path coefficients, a × b products and BCa 95% CI for these products were presented in Table 4. First, the associations between occupational stress evaluated by the ERI scale and depersonalisation (the c path) were determined. Positive associations of extrinsic effort and overcommitment with depersonalisation and a negative association of reward with depersonalisation were observed among both male and female bank employees. The mediating role of PsyCap on the association between occupational stress and depersonalisation was then estimated.

For male bank employees, extrinsic effort was significantly and negatively associated with PsyCap while reward showed a significant positive association, but overcommitment was not significantly associated with PsyCap (the a path). After controlling for the predictor variables, PsyCap was significantly and negatively associated with depersonalisation (the b path). Thus, PsyCap significantly mediated the associations of both extrinsic effort and reward with depersonalisation. When PsyCap was included in the model as a mediator, the direct pathway between extrinsic effort and depersonalisation remained significant but that between reward and depersonalisation was not significant (the c’ path).

For female bank employees, extrinsic effort and overcommitment were negatively and reward was positively associated with PsyCap (the a path). Consistent with the results for male bank employees, PsyCap was significantly and negatively associated with depersonalisation after controlling for extrinsic effort, reward and overcommitment. Thus, PsyCap significantly mediated the associations of extrinsic effort, reward and overcommitment with depersonalisation. The direct pathways between extrinsic effort, reward, overcommitment and depersonalisation (the c’ path) remained significant when PsyCap was included in the model as a mediator.

To estimate the effect size of the mediating pathway, we calculated the proportion of the total effect of the independent variable on the dependent variable (c) that was mediated by PsyCap using the formula (a × b)/c. For male bank employees, the proportions of PsyCap mediation were 14.06% for extrinsic effort, and 38.56% for reward. For female bank employees, the proportions of PsyCap mediation were 17.85% for extrinsic effort, 34.50% for reward and 32.15% for overcommitment.

**Table 4:** Regression analysis results, with depersonalisation as outcome and psychological capital as mediator.

Predictors	Path Coefficients				a × b (BCa 95% CI)	R^2^
	c	a	b	c’		
Males						
Extrinsic effort	0.334 **	‒0.228 **	‒0.206 **	0.287 **	0.047 (0.022, 0.083)	0.140
Reward	‒0.144 **	0.223 **	‒0.249 **	‒0.088	‒0.056 (‒0.098,‒0.028)	0.071
Overcommitment	0.153 **	‒0.056	‒0.259 **	0.139 **	0.015 (‒0.012, 0.043)	0.081
Females						
Extrinsic effort	0.314 **	‒0.286 **	‒0.196 **	0.258 **	0.056 (0.033, 0.087)	0.132
Reward	‒0.169 **	0.237 **	‒0.246 **	‒0.111 **	‒0.059 (‒0.087, ‒0.037)	0.083
Overcommitment	0.129 **	‒0.162 **	‒0.256 **	0.087 **	0.042 (0.021, 0.069)	0.080

Notes: c: associations of extrinsic effort, reward and overcommitment with depersonalization; a: associations of extrinsic effort, reward and overcommitment with PsyCap; b: association between PsyCap and depersonalization after controlling for the predictor variables; c’: associations of extrinsic effort, reward and overcommitment with depersonalization after adding PsyCap as mediator; a × b: the product of a and b; BCa 95% CI: the bias-corrected and accelerated 95% confidence interval age, marital status, education and weekly work time are covariates; ** significant at the 0.01 level (2-tailed).

### 3.5. Regression Analysis with Professional Accomplishment as the Criterion Variable

Path coefficients, a × b products and BCa 95% CI for these products were presented in Table 5. First, the associations between occupational stress evaluated by the ERI scale and professional accomplishment (the c path) were determined. A positive association of effort with professional accomplishment was observed among both male and female bank employees. The mediating role of PsyCap on the association between occupational stress and professional accomplishment was then estimated.

For male bank employees, extrinsic effort and overcommitment were not significantly associated with professional accomplishment (the c and c’ paths). After controlling for the predictor variables, PsyCap was not significantly associated with professional accomplishment (the b path). As a result, PsyCap did not mediate the associations of extrinsic effort, reward and overcommitment with professional accomplishment found in our study.

As for the results of male bank employees, PsyCap did not mediate the associations of extrinsic effort and overcommitment with professional accomplishment for female bank employees. However, reward was positively significantly associated with PsyCap (the a path). After controlling for the predictor variables, PsyCap was significantly and negatively associated with professional accomplishment (the b path). PsyCap thus significantly mediated the association between reward and professional accomplishment. The direct pathway between reward and professional accomplishment remained significant (the c’ path) when PsyCap was included in the model as a mediator.

To estimate the effect size of the mediating pathway, we calculated the proportion of the total effect of the independent variable on the dependent variable (c) that was mediated by PsyCap using the formula (a × b)/c. For female bank employees, the proportion of PsyCap mediation was 17.12% for reward.

**Table 5:** Regression analysis results, with professional accomplishment as outcome and psychological capital as mediator.

Predictors	Path Coefficients				a × b (BCa 95% CI)	R^2^
	c	a	b	c’		
Males						
Extrinsic effort	0.038	‒0.228 **	0.087	0.058	‒0.019 (‒0.045,‒0.003)	0.051
Reward	0.156 **	0.223 **	0.048	0.146 **	0.011 (‒0.007, 0.034)	0.066
Overcommitment	0.003	‒0.056	0.076	0.007	‒0.005 (‒0.019, 0.002)	0.048
Females						
Extrinsic effort	‒0.003	‒0.286 **	0.192 **	0.052	‒0.055 (‒0.081, ‒0.033)	0.062
Reward	0.191 **	0.237 **	0.138 **	0.158 **	0.033 (0.015, 0.054)	0.083
Overcommitment	‒0.023	‒0.162 **	0.177 **	0.005	‒0.029 (‒0.048, ‒0.015)	0.060

Notes: c: associations of extrinsic effort, reward and overcommitment with professional accomplishment; a: associations of extrinsic effort, reward and overcommitment with PsyCap; b: association between PsyCap and professional accomplishment after controlling for the predictor variables; c’: associations of extrinsic effort, reward and overcommitment with professional accomplishment after adding PsyCap as mediator; a × b: the product of a and b; BCa 95% CI: the bias-corrected and accelerated 95% confidence interval age, marital status, education and weekly work time are covariates; ** significant at the 0.01 level (2-tailed).

## 4. Discussion

In this study, we examine the relationship between occupation stress and job burnout, as well as the mediating effect of PsyCap in this relationship among Chinese bank employees. The large sample from Liaoning Province had a high effective response rate (77.8%) which seemed to be able to provide a good representation of our study population and increase the generalization of the conclusion in our study.

The levels of emotional exhaustion and personal accomplishment in both male and female bank employees in our sample were lower than in doctors (male individuals: 12.93 ± 7.21, 23.03 ± 9.39; female individuals: 13.51 ± 7.22, 23.14 ± 8.72) and nurses (11.74 ± 7.14, 23.34 ± 9.60) in China, while the level of depersonalisation was higher (male doctors: 7.74 ± 6.06; female doctors: 7.61 ± 6.01; nurses: 7.12 ± 5.67) [4,14]. The average scores of the three dimensions of job burnout in our sample were higher than in five occupational groups (managers, clerks, foremen, technical professionals, and blue-collar workers) from three nations (Finland, Sweden and The Netherlands) [50]. Compared with Japanese doctors (95% CI: 8.58–9.41), only the level of personal accomplishment was slightly higher in this case [51].

In the present study, we found that the correlations of the three dimensions of occupational stress in the three dimensions of job burnout among male bank employees were the same as for female bank employees. Extrinsic effort and overcommitment were positively related to emotional exhaustion and depersonalisation, respectively. Meanwhile, reward was negatively related to emotional exhaustion and depersonalisation, and positively related to personal accomplishment. These results corresponded with those of previous studies on different vocational workers [17,18,19,52]. According to the authors, bank employees with higher levels of extrinsic effort and overcommitment spend too much time and energy accomplishing their performance goals and dealing with customer dissatisfaction. Such employees lack the time to relieve the competitive pressures they face from being laid off and policy reform. However, bank employees, being engaged in a service profession, rarely receive praise, whether from customers or their superiors. These factors may contribute to their high emotional exhaustion and depersonalisation and low personal accomplishment. Therefore, measures for controlling occupational stress should be introduced in job burnout interventions for Chinese bank employees. Bank administrators should try to reduce workload and working hours, and to provide appropriate awards based on individual ability [46]. Bank employees also require a cultural and ethical work atmosphere.

As a positive personal resource, PsyCap can help employees cope with occupational stressors and work more efficiently. It has been suggested that PsyCap can predict positive work attitudes and outcomes. For example, PsyCap can enhance the levels of job satisfaction and job performance [29,30,31,32]. PsyCap is also a highly effective resource that can decrease job stress, burnout, and depression [14,25,35]. Our results showed that PsyCap was significantly and negatively correlated with emotional exhaustion and depersonalisation, and significantly and positively correlated with personal accomplishment among both male and female bank employees. Therefore, bank employees, with adequate PsyCap, are less likely to feel tired and their burnout symptoms tend to be alleviated. This implied that PsyCap might significantly affect job burnout and be a positive resource to combat job burnout among bank employees in China.

To our knowledge, our research is the first to explore the mediating role of PsyCap in the relationship between occupational stress and job burnout in China. A sex difference was observed in PsyCap mediation of the association between occupational stress and job burnout. For male bank employees, PsyCap mediated the effects of the two dimensions of occupational stress (extrinsic effort and reward) on the two dimensions of job burnout (emotional exhaustion and depersonalisation). Meanwhile, for female bank employees, PsyCap partially mediated the effects of the three dimensions of occupational stress (extrinsic effort, reward and overcommitment) on the two dimensions of job burnout (emotional exhaustion and depersonalisation). PsyCap also mediated between reward and personal accomplishment. Various reasons for this gender difference should be considered. Female individuals may simply have higher perceptual sensitivity than male individuals, and may pay more attention to interpersonal relationships and working atmosphere. In terms of work engagement, female individuals expend more energy and emotion than male individuals. Therefore, female bank employees with more overcommitment are more likely to experience lower PsyCap, leading in turn to emotional exhaustion and depersonalisation. Having different focuses with male individuals, female individuals care more about praise or encouragement than material rewards. If they have higher levels of reward and PsyCap, female bank employees will feel higher personal accomplishment. Consequently, it is suggested that future research should focus on gender differences to investigate appropriate strategies and practices for PsyCap development.

Besides providing additional support for previous findings, the main contribution of our study is to highlight that occupational stress may affect the risk of job burnout in bank employees via a mediating mechanism of PsyCap. The study results identified PsyCap as a positive resource for reducing the negative effects of occupational stress on job burnout. Given limited resources, besides decreasing employees’ stress, we can prevent and relieve job burnout through improving employees’ PsyCap [38]. For instance, bank managers should communicate effectively with employees, establish fair and impartial systems, or set appropriate and challenging job goals to boost employees’ confidence. Bank employees’ personal qualities and working ability can also be enhanced through on-site training. Hence, intervention strategies previously developed to enhance PsyCap level should be applied to bank staff in China.

## 5. Conclusions

Among both male and female bank employees, extrinsic effort and overcommitment were positively related to emotional exhaustion and cynicism, while reward was negatively related to emotional exhaustion and depersonalisation and positively related to personal accomplishment. For male bank employees, PsyCap respectively mediated the effects of the two dimensions of occupational stress (extrinsic effort and reward) on the two dimensions of job burnout (emotional exhaustion and depersonalisation). For female bank employees, PsyCap partially mediated the effects of the three dimensions of occupational stress (extrinsic effort, reward and overcommitment) on the two dimensions of job burnout (emotional exhaustion and depersonalisation), and also partially mediated between the one dimension of occupational stress (reward) and the one dimension of job burnout (personal accomplishment). Therefore, besides reducing excessive effort and overcommitment and increasing resource rewards, bank administrators can increase PsyCap to decrease job burnout among bank employees, especially female individuals.

Despite making some contributions as outlined above, this study suffers several limitations. First, it used a cross-sectional design, making it impossible to draw any causal relations among occupational stress, PsyCap and job burnout. In future, the study results thus should be confirmed through a prospective study. Second, data were obtained by self-reports, which can introduce bias. Participants may have underestimated or overestimated the relationship between occupational stress and job burnout. Third, the study population was limited to staff from state-owned banks. Private and municipal banks were not considered [41]. Further research should also consider employees of private and municipal banks. Given these limitations, it is necessary to be prudent in presenting and interpreting the test results.

## References

[1] Maslach C., Schaufeli W.B., Leiter M.P. (2001). Job burnout. Ann. Rev. Psychol..

[2] Schaufeli W.B., Leiter M.P., Maslach C., Jackson S.E. (1996). Maslach Burnout Inventory.

[3] Freudenberger H.J. (1974). Staff burnout. J. Soc. Issues..

[4] Wu H., Liu L., Sun W., Zhao X., Wang J., Wang L. (2014). Factors related to burnout among Chinese female hospital nurses: Cross-sectional survey in Liaoning Province of China. J. Nurs. Manag..

[5] Cooke G.P.E., Doust J.A., Steele M.C. (2013). A survey of resilience, burnout, and tolerance of uncertainty in Australian general practice registrars. BMC Med. Educ..

[6] Khamisa N., Peltzer K., Oldenburg B. (2013). Burnout in relation to specific contributing factors and health outcomes among nurses: A systematic review. Int. J. Environ. Res. Public Health.

[7] Lindwall M., Gerber M., Jonsdottir I.H., Börjesson M., Ahlborg G. (2014). The relationships of change in physical activity with change in depression, anxiety, and burnout: A longitudinal study of Swedish healthcare workers. Health Psychol..

[8] Ding Y.W., Qu J.W., Yu X.S., Wang S. (2014). The mediating effects of burnout on the relationship between anxiety symptoms and occupational stress among community healthcare workers in China: A cross-sectional study. PLoS One.

[9] Mohren D.C.L., Swaen G.M.H., Kant I.J., van Amelsvoort L.G., Borm P.J., Galama J.M. (2003). Common infections and the role of burnout in a Dutch working population. J. Psychosom. Res..

[10] Lin Q.H., Jiang C.Q., Lam T.H. (2013). The relationship between occupational stress, burnout, and turnover intention among managerial staff from a sino-japanese joint venture in Guangzhou, China. J. Occup. Health.

[11] Gorji M., Vaziri S. (2011). The survey job burnout status and its relation with the performance of the employees (Case study: Bank). Int. Proc. Econ. Dev. Res..

[12] Jasperse M., Herst P., Dungey G. (2014). Evaluating stress, burnout and job satisfaction in New Zealand radiation oncology departments. Eur. J. Cancer Care.

[13] Wang Y., Liu L., Wang J., Wang L. (2012). Work-family conflict and burnout among Chinese doctors: The mediating role of psychological capital. J. Occup. Health.

[14] Zhang L., Zhao J., Xiao H., Zheng H., Xiao Y., Chen M., Chen D. (2014). Mental health and burnout in primary and secondary school teachers in the remote mountain areas of Guangdong Province in the People’s Republic of China. Neuropsychiatr. Dis. Treat..

[15] Yom Y.H. (2013). Analysis of burnout and job satisfaction among nurses based on the job demand-resource model. J. Korean Acad. Nurs..

[16] Finney C., Stergiopoulos E., Hensel J., Bonato S., Dewa C.S. (2013). Organizational stressors associated with job stress and burnout in correctional officers: A systematic review. BMC Public Health.

[17] Wu S., Zhu W., Li H., Wang Z., Wang M. (2008). Relationship between job burnout and occupational stress among doctors in China. Stress Health.

[18] Bagaajav A., Myagmarjav S., Nanjid K., Otgon S., Chae Y.M. (2011). Burnout and job stress among Mongolian doctors and nurses. Ind. Health.

[19] Poghosyan L., Aiken L.H., Sloane D.M. (2009). Factor structure of the Maslach burnout inventory: An analysis of data from large scale cross-sectional surveys of nurses from eight countries. Int. J. Nurs. Stud..

[20] Yan S.Y., He Y.P., Lai S.R., Ding X.C., Yang P.D., Dai D.M. (2011). Association between occupational stress and job burnout among staff in companies. J. Environ. Occup. Med..

[21] Wang Y., Ramos A., Wu H., Liu L., Yang X., Wang J., Wang L. (2014). Relationship between occupational stress and burnout among Chinese teachers: A cross-sectional survey in Liaoning, China. Int. Arch. Occup. Environ. Health.

[22] Silva J.L., Navarro V.L. (2012). Work organization and the health of bank employees. Lat. Am. J. Nurs..

[23] Shahram H., Somayeh A., Behnam G.F. (2014). The effect of occupational stress, psychological stress and burnout on employee performance: Evidence from banking industry. Manag. Sci. Lett..

[24] Ozkan A., Ozdevecioğlu M. (2013). The effects of occupational stress on burnout and life satisfaction: A study in accountants. Qual. Quant..

[25] Zhao J., Zhang X.C. (2010). Work stress and job burnout: The moderating effects of psychological capital. J. Henan Norm. Univ. (Nat. Sci.).

[26] Luthans F., Avolio B.J., Walumbwa F.O., Li W.X. (2005). The psychological capital of Chinese workers: Exploring the relationship with performance. Manag. Organ. Rev..

[27] Luthans F., Youssef C.M. (2004). Human, social, and now positive psychological management: Investing in people for competitive advantage. Organ. Dyn..

[28] Avey J.B., Luthans F., Youssef C.M. (2010). The additive value of positive psychological capital in predicting work attitudes and behaviors. J. Manag..

[29] Fu J., Sun W., Wang Y., Yang X., Wang L. (2013). Improving job satisfaction of Chinese doctors: The positive effects of perceived organizational support and psychological capital. Public Health.

[30] Sun T., Zhao X.W., Yang L.B., Fan L.H. (2012). The impact of psychological capital on job embeddedness and job performance among nurses: A structural equation approach. J. Adv. Nurs..

[31] Walumbwa F.O., Peterson S.J., Avolio B.J., Hartnell C.A. (2010). An investigation of the relationships among leader and follower psychological capital, service climate, and job performance. Pers. Psychol..

[32] Peng J., Jiang X., Zhang J., Xiao R., Song Y., Feng X., Zhang Y., Miao D. (2013). The impact of psychological capital on job burnout of chinese nurses: The mediator role of organizational commitment. PLoS One.

[33] Nguyen T.D., Nguyen T.T.M. (2012). Psychological capital, quality of work life, and quality of life of marketers: Evidence from Vietnam. J. Macromarketing.

[34] Fariborz R., Ahmadreza K.M., Zahra M. (2013). Emotional mediators of psychological capital on well-being: The role of stress, anxiety, and depression. Manag. Sci. Lett..

[35] Avey J.B., Luthans F., Jensen S.M. (2009). Psychological capital: A positive resource for combating employee stress and turnover. Human Resour. Manag..

[36] Rego A., Sousa F., Marques C. (2012). Authentic leadership promoting employees’ psychological capital and creativity. J. Bus. Res..

[37] Luthans F., Norman S.M., Avolio B.J., Avey J.B. (2008). The mediating role of psychological capital in the supportive organizational climate-employee performance relationship. J. Organ. Behav..

[38] Wang Y., Chang Y., Fu J., Wang L. (2012). Work-family conflict and burnout among Chinese female nurses: The mediating effect of psychological capital. BMC Public Health.

[39] Wu H., Zhao Y., Wang J.N., Wang L. (2010). Factors associated with occupational stress among Chinese doctors: A cross-sectional survey. Int. Arch. Occup. Environ. Health.

[40] Hassan E.M. (2009). Gender, self-concept and occupational status differentials in occupational stress among bank workers in Lagos State. Soc. Sci..

[41] Yang W.J., Li J. (2004). Measurement of psychosocial factors in work environment: Application of two models of occupational stress. Chinese J. Ind. Hyg. Occup. Dis..

[42] Xu W., Yu H., Gao W., Guo L., Zeng L., Zhao Y. (2011). When job stress threatens Chinese workers: Combination of job stress models can improve the risk estimation for coronary heart disease-The BADCAR study. J. Occup. Environ. Med..

[43] Liu L., Chang Y., Fu J., Wang J., Wang L. (2012). The mediating role of psychological capital on the association between occupational stress and depressive symptoms among Chinese physicians: A cross-sectional study. BMC Public Health.

[44] Luthans F., Avolio B.J., Avey J.B., Norman S.M. (2007). Positive psychological capital: Measurement and relationship with performance and satisfaction. Pers. Psychol..

[45] Pan Q.Q., Zhou Z.K. (2009). Relationships among psychological capital, coping style and mental health of impoverished college students. China J. Health Psychol..

[46] Maslach C., Jackson S.E. (1981). The measurement of experienced burnout. J. Organ. Behav..

[47] Li J., Yang W., Cheng Y., Sieqrist J., Cho S.I. (2005). Effort-reward imbalance at work and job dissatisfaction in Chinese healthcare workers: A validation study. Int. Arch. Occup. Environ. Health.

[48] Mantelou E., Tzioti M.C., Degleris N.E., Solias A., Karamberi M. (2010). Job burnout, self-efficacy theory and job satisfaction in a sample of greek bank clerks. Ann. Gen. Psychiatry..

[49] Preacher K.J., Hayes A.F. (2008). Asymptotic and resampling strategies for assessing and comparing indirect effects in multiple mediator models. Behav. Res. Meth..

[50] Schutte N., Toppinen S., Kalimo R., Schaufeli W. (2000). The factorial validity of the Maslach Burnout Inventory-General Survey (MBI-GS) across occupational groups and nations. J. Occup. Organ. Psychol..

[51] Saijo Y., Chiba S., Yoshioka E., Kawanishi Y., Nakaqi Y., Ito T., Suqioka Y., Kitaoka-Hiqashiquchi K., Yoshida T. (2013). Job stress and burnout among urban and rural hospital physicians in Japan. Aust. J. Rural Health.

[52] Khattak J.K., Khan M.A., Haq A.U., Arif M., Minhas A.A. (2011). Occupational stress and burnout in Pakistan’s banking sector. Afr. J. Bus. Manag..

